# The role of Rabconnectin3a in cilia length regulation

**DOI:** 10.1186/2046-2530-4-S1-P70

**Published:** 2015-07-13

**Authors:** B Tavares, P Pintado, SS Lopes

**Affiliations:** 1 CEDOC, Chronic Diseases Research Center, NOVA Medical School / Faculdade de Ciências Médicas, Universidade Nova de Lisboa, Campo Dos Mártires Da Pátria, 130, 1169-056 Lisbon, Portugal

## Background

Using the zebrafish mutant for the *deltaD *gene (*dld^-/-^*), it was shown the involvement of Notch signaling in the control of cilia length in the cells of the fish laterality organ (Kupffer's Vesicle, KV) [1]. Further research based on KV specific microarray screening allowed the discovery of several genes with differential expression. Of these, 23% were associated with ciliogenesis and upon analysis, many proved to be involved in cellular trafficking.

*Rabconnectin3a *or *rbcn3a *was strongly downregulated in *dld^-/- ^*KV cells. Homologs of this gene have been associated with Notch signaling in Drosophila and mammalian cells through the regulation of the V-ATPase activity [2,3]. *Rbcn3a *had also been associated with vesicular acidification in zebrafish hair cells [4] and with vesicular endocytosis and maturation in zebrafish neural crest migration [5].

## Objective

We investigated the role of Rbcn3a in cilia length regulation.

## Methods

We used a Morpholino against *rbcn3a *and fluorescent confocal imaging to explore cilia length. Furthermore we observed the consequences of reduced Rbcn3a in organ *situs *by ISH. We also performed rescue experiments by injecting *rbcn3a *full length mRNA at 1-cell stage *dld^-/- ^*KO mutants.

## Results

We showed that the downregulation of *rbcn3a *negatively regulates cilia length and that this can be rescued by *rbcn3a *overexpression in *dld^-/- ^*embryos.

## Conclusion

The ciliary phenotype in *dld^-/- ^*mutants is partially due to the downregulation of *rbcn3a*. Our hypothesis is that a generalized decrease in endocytic acidification, by deregulating the V-ATPase activity, results in shorter cilia.
